# Characterizing the relationships between tertiary and community cancer providers: Results from a survey of medical oncologists in Southern California

**DOI:** 10.1002/cam4.4119

**Published:** 2021-07-31

**Authors:** Nicholas J. Salgia, Alexander Chehrazi‐Raffle, JoAnn Hsu, Zeynep Zengin, Sabrina Salgia, Neal S. Chawla, Luis Meza, Jasnoor Malhotra, Nazli Dizman, Ramya Muddasani, Nora Ruel, Mary Cianfrocca, Jun Gong, Sidharth Anand, Victor Chiu, James Yeh, Sumanta K. Pal

**Affiliations:** ^1^ Department of Medical Oncology & Experimental Therapeutics City of Hope Comprehensive Cancer Center Duarte CA USA; ^2^ Department of Medicine City of Hope Comprehensive Cancer Center Duarte CA USA; ^3^ Biostatistics and Mathematical Modeling Core City of Hope Comprehensive Cancer Center Duarte CA USA; ^4^ Division of Hematology/Oncology Department of Medicine Cedars‐Sinai Medical Center Los Angeles CA USA; ^5^ Division of Hematology/Oncology David Geffen School of Medicine at UCLA Los Angeles CA USA; ^6^ Division of Hematology/Oncology UCLA‐Olive View Medical Center Los Angeles CA USA; ^7^ Division of Hematology and Medical Oncology Department of Medicine Harbor‐UCLA Medical Center Torrance CA USA

**Keywords:** Clinical trials, community oncology, referrals, tertiary cancer center

## Abstract

**Background:**

Tertiary cancer centers offer clinical expertise and multi‐modal approaches to treatment alongside the integration of research protocols. Nevertheless, most patients receive their cancer care at community practices. A better understanding of the relationships between tertiary and community practice environments may enhance collaborations and advance patient care.

**Methods:**

A 31‐item survey was distributed to community and tertiary oncologists in Southern California using REDCap. Survey questions assessed the following attributes: demographics and features of clinical practice, referral patterns, availability and knowledge of clinical trials and precision medicine, strategies for knowledge acquisition, and integration of community and tertiary practices.

**Results:**

The survey was distributed to 98 oncologists, 85 (87%) of whom completed it. In total, 52 (61%) respondents were community practitioners and 33 (38%) were tertiary oncologists. A majority (56%) of community oncologists defined themselves as general oncologists, whereas almost all (97%) tertiary oncologists reported a subspecialty. Clinical trial availability was the most common reason for patient referrals to tertiary centers (73%). The most frequent barrier to tertiary referral was financial considerations (59%). Clinical trials were offered by 97% of tertiary practitioners compared to 67% of community oncologists (*p* = 0.001). Most oncologists (82%) reported only a minimal‐to‐moderate understanding of clinical trials available at regional tertiary centers.

**Conclusions:**

Community oncologists refer patients to tertiary centers primarily with the intent of clinical trial enrollment; however, significant gaps exist in their knowledge of trial availability. Our results identify the need for enhanced communication and collaboration between community and tertiary providers to expand patients’ access to clinical trials.

## Graphical abstract

Further relationships between community and tertiary medical oncologists are imperative to increasing clinical trial enrollment and successful patient outcomes. This study reports significant gaps in the knowledge of clinical trial availability among oncologists and identifies a need for increased communication and collaboration between the practice settings.

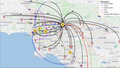

## 1 INTRODUCTION

Tertiary cancer centers offer a multi‐modal approach to cancer diagnosis and care, incorporating experts in various cancer‐related disciplines. The National Cancer Institute‐designated Comprehensive Cancer Center (NCI‐CCC) model aims to distinguish tertiary cancer centers with expertise in clinical cancer care and basic/translational cancer biology research. There are 51 NCI‐CCCs throughout the United States, with Southern California hosting 5 such centers as well as 2 NCI‐designated Basic Laboratory Cancer Centers.^1^ From 1998 to 2008, 6.4% of adults diagnosed with cancer in Los Angeles County (the most populous county in California) received care at an NCI‐CCC.^2^ The authors of this study reported that patients who received their first line of treatment at an NCI‐CCC had more favorable clinical outcomes than those who received care at non‐NCI‐CCC practices.

Tertiary cancer centers, particularly those designated as NCI‐CCCs, have long been leaders of clinical research, providing patients with access to experimental therapies through clinical trials that are often unavailable in the community setting. The clinical trial paradigm for the implementation and approval of new therapeutic options in oncology has been well‐established.^3^ However, patient participation in clinical trials remains at approximately 5%, in part due to low trial enrollment from community practices.^4^ Previous studies have identified several barriers to trial accrual in the community setting, including transportation and out‐of‐network costs for patients and lack of trial knowledge, lack of trial infrastructure, and lack of time for community physicians.^5^, ^6^, ^7^, ^8^, ^9^ These discordances may be just one facet of a broader disconnect between community and tertiary oncology practices.

The greater Southern California region, given its density of tertiary cancer centers (including NCI‐CCCs and other academic centers) and its large volume of tertiary and community oncologists, provides an outsized opportunity to assess the relationships between community and tertiary oncology practices. We sought to investigate the characteristics of clinical practice at tertiary and community oncology practices. We additionally examine provider perspectives on the engagement between community practices and tertiary centers through a survey distributed to Southern California oncologists.

## 2 MATERIALS AND METHODS

### 2.1 Survey participants

Medical oncologists practicing in Southern California were invited by email to participate in an online survey. Oncologists were identified through: (1) a retrospective review of referring providers to the authors’ institution, (2) review of faculty lists at tertiary centers, and (3) a curated database of previous clinical fellows at the authors’ institutions. Tertiary providers were defined as medical oncologists who spend a majority of their clinical hours at an NCI‐CCC or at an academic medical center with a stand‐alone cancer center as part of the hospital system. Community providers were defined as oncologists who spend the majority of their clinical hours at either (1) a community hospital without a stand‐alone cancer center, (2) an outpatient community practice either affiliated or not affiliated with a tertiary center, or (3) a public hospital (including Veterans Affairs and Los Angeles Department of Health Services institutions).

### 2.2 Provider survey

A link to the survey was provided to oncologists within the email invitation. This survey contained 31 questions, of which 24 were multiple choice, 3 were fill‐in‐the‐blank, 1 was a ranking, and 3 were open‐ended questions. The survey is provided in its entirety in Appendix 1. The following attributes of the provider and his or her practice were interrogated in this survey: demographics and features of clinical practice, referral patterns, availability and knowledge pertaining to clinical trials and precision medicine, strategies for knowledge acquisition, and integration of community and tertiary practices. It was estimated that the survey would take approximately 8–10 minutes to complete. The content of the survey and distribution to the aforementioned audience were approved by the City of Hope Comprehensive Cancer Center institutional review board.

### 2.3 Data collection and statistical analysis

Survey responses were collected through REDCap, a secure online data repository platform.^10^ Participant email addresses were collected alongside survey responses. Only responses from individuals who had completed the survey in its entirety were included for analysis. Results were summarized using counts and percentages, and stratified by type of oncologist (community hospital vs. tertiary care). Comparisons between community and tertiary oncologists were calculated using a two sided chi‐square test. SAS^®^ 9.4 was used for all calculations and graphs, and 0.05 was used as a threshold for statistical significance.

## 3 RESULTS

### 3.1 Survey distribution, provider demographics, and features of clinical practice

The survey was distributed to 98 oncologists in Southern California, of which 85 (87%) completed the survey in its entirety. Of those who completed the survey, 33 were tertiary center oncologists (39%) and 52 were community practitioners (61%). A larger proportion of respondents in the community were male. More than one fifth of community oncologists were not affiliated with a tertiary oncology center. The largest proportion of respondents had been in clinical practice for <5 years (39%) and saw between 41 and 60 patients in clinic per week (33%). Areas of specialty were diverse, with the most frequent being breast (19%), gastrointestinal (12%), and genitourinary (7%) oncology among all respondents. A much larger proportion of community hospital oncologists delineated no disease‐related specialization. Twenty‐nine of the 30 respondents (97%) who classified themselves as general medical oncologists without a specialty were community oncologists, whereas only 1 was a tertiary center physician (3%). The respondents perceived sarcomas (42%), central nervous system malignancies (27%), and gynecologic cancers (11%) as their greatest areas of weakness. Full demographics and characteristics of clinical practice for the cohort are provided in Table 1.

**TABLE 1:** Provider demographics and clinical practice features

	Community hospital oncologists (*n* = 52)	Tertiary care oncologists (*n* = 33)	*p* value	All responders (*n* = 85)
Gender				
Female	16 (30.8%)	18 (54.5%)	0.03	34 (40.0%)
Male	36 (69.2%)	15 (45.5%)		51 (60.0%)
Age				
31–40	24 (46.2%)	17 (51.5%)	0.9	41 (48.2%)
41–50	18 (34.6%)	9 (27.3%)		27 (31.8%)
51–60	5 (9.6%)	4 (12.1%)		9 (10.6%)
61–70	2 (3.8%)	2 (6.1%)		4 (4.7%)
71–80	3 (5.8%)	1 (3.0%)		4 (4.7%)
Affiliated tertiary cancer center				
Cedars‐Sinai	4 (7.7%)	3 (9.1%)	0.03	7 (8.2%)
City of Hope	30 (57.7%)	21 (63.6%)		51 (60.0%)
UCLA	5 (9.6%)	6 (18.2%)		11 (12.9%)
USC	1 (1.9%)	3 (9.1%)		4 (4.7%)
No affiliation	12 (23.1%)	0 (0.0%)		12 (14.1%)
Years as a medical oncology attending				
<5	19 (36.5%)	14 (42.4%)	0.8	33 (38.8%)
6–10	13 (25.0%)	7 (21.2%)		20 (23.5%)
11–15	8 (15.4%)	4 (12.1%)		12 (14.1%)
16–20	4 (7.7%)	3 (9.1%)		7 (8.2%)
≥21	8 (15.4%)	5 (15.1%)		13 (15.3%)
Years affiliated with current practice site				
<5	36 (69.2%)	19 (57.6%)	0.6	55 (64.7%)
6–10	12 (23.1%)	8 (24.2%)		20 (24.0%)
11–15	2 (3.8%)	2 (6.1%)		4 (4.7%)
16–20	1 (1.9%)	3 (9.1%)		4 (4.7%)
≥21	1 (1.9%)	1 (3.0%)		2 (2.4%)
Area of specialty				
Yes‐			<0.0001	
Breast	8 (15.4%)	8 (24.2%)		16 (18.8%)
Gastrointestinal	5 (9.6%)	5 (15.2%)		10 (11.8%)
Genitourinary	2 (3.8%)	4 (12.1%)		6 (7.1%)
Gynecologic	0 (0.0%)	1 (3.0%)		1 (1.2%)
Head and neck	0 (0.0%)	2 (6.1%)		2 (2.4%)
Thoracic	4 (7.7%)	3 (9.1%)		7 (8.2%)
Other	0 (0.0%)	8 (24.2%)		8 (9.4%)
No ‐ General oncology	29 (55.8%)	1 (3.0%)		30 (35.3%)
Unclassified	4 (7.7%)	1 (1.2%)		5 (5.9%)
Resources most used to learn about current clinical research				
Local or online CME events	1 (1.9%)	0 (0.0%)	<0.0001	1 (1.2%)
Online tools	37 (71.2%)	7 (21.2%)		44 (51.8%)
Professional meetings (i.e., ASCO)	11 (21.2%)	17 (51.5%)		28 (32.9%)
PubMed or other literature databases	3 (5.8%)	9 (27.3%)		12 (14.1%)
Weekly clinic volume				
<10	2 (3.8%)	1 (3.0%)	0.04	3 (3.5%)
11–20	1 (1.9%)	4 (12.1%)		5 (5.9%)
21–40	9 (17.3%)	11 (33.3%)		20 (23.5%)
41–60	16 (30.8%)	12 (36.4%)		28 (32.9%)
61–80	15 (28.8%)	4 (12.1%)		19 (22.4%)
≥81	8 (15.4%)	1 (3.0%)		9 (10.6%)
Unclassified	1 (1.9%)	0 (0.0%)		1 (1.2%)
Number of annual referrals to tertiary centers				
None	2 (3.8%)	3 (9.1%)	0.2	5 (5.9%)
1–5	22 (42.3%)	19 (57.6%)		39 (45.9%)
6–10	16 (30.8%)	9 (27.3%)		25 (29.4%)
11–20	8 (15.4%)	2 (6.1%)		10 (11.8%)
≥21	5 (9.6%)	0 (0.0%)		5 (5.9%)
Not disclosed	1 (1.9%)	0 (0.0%)		1 (1.2%)
Insurance a factor when deciding on referrals to tertiary centers	25 (48.1%)	5 (15.2%)	0.003	30 (35.3%)
Offering clinical trials in clinical practice	35 (67.3%)	32 (97.0%)	0.001	67 (78.8%)
Genomic profiling platform used most often				
Ashion GEM Extra	2 (3.8%)	14 (42.4%)	<0.0001	16 (18.8%)
Caris	7 (13.5%)	2 (6.1%)		9 (10.6%)
FoundationOne	24 (46.2%)	1 (3.0%)		25 (29.4%)
Guardant360	2 (3.8%)	2 (6.1%)		4 (4.7%)
Tempus	6 (11.5%)	9 (27.3%)		15 (17.6%)
Other	10 (19.2%)	5 (15.2%)		15 (17.6%)
Do not offer genomic profiling	1 (1.9%)	0 (0.0%)		1 (1.2%)
Primary reason to referring to other tertiary centers				
Clinical trial available at specific institution	32 (61.5%)	30 (90.9%)	0.01	62 (72.9%)
Patient requests	2 (3.8%)	2 (6.1%)		4 (4.7%)
Patient transportation needs	2 (3.8%)	1 (3.0%)		3 (3.5%)
Physician expertise	14 (26.9%)	0 (0.0%)		14 (16.5%)
Not disclosed	2 (3.8%)	0 (0.0%)		2 (2.4%)
Biggest barrier getting patients seen at other tertiary centers				
Financial considerations	32 (61.5%)	18 (54.6%)	0.03	50 (58.8%)
Lengthy wait times for providers	5 (9.6%)	10 (30.3%)		15 (17.6%)
Transportation to tertiary campus	13 (25.0%)	4 (12.1%)		17 (20.0%)
Not disclosed	2 (3.8%)	1 (3.0%)		3 (3.5%)
Characteristics of patients referred to tertiary centers^a^				
Early stage disease	0 (0.0%)	3 (9.1%)	0.05	3 (3.6%)
Advanced disease, treatment‐naïve	2 (4.0%)	0 (0.0%)		2 (2.4%)
Advanced disease, treatment‐refractory	48 (96.0%)	30 (90.9%)		78 (94.0%)
Phase of study most often referred to^a^				
Phase I	14 (31.1%)	20 (60.6%)	0.003	34 (43.6%)
Phase II	14 (31.1%)	11 (33.3%)		25 (32.1%)
Phase III	17 (37.8%)	2 (6.1%)		19 (24.4%)

^a^ Only providers who referred ≥1 patient per year included in calculations.

### 3.2 Referrals to tertiary centers

**FIGURE 1:**
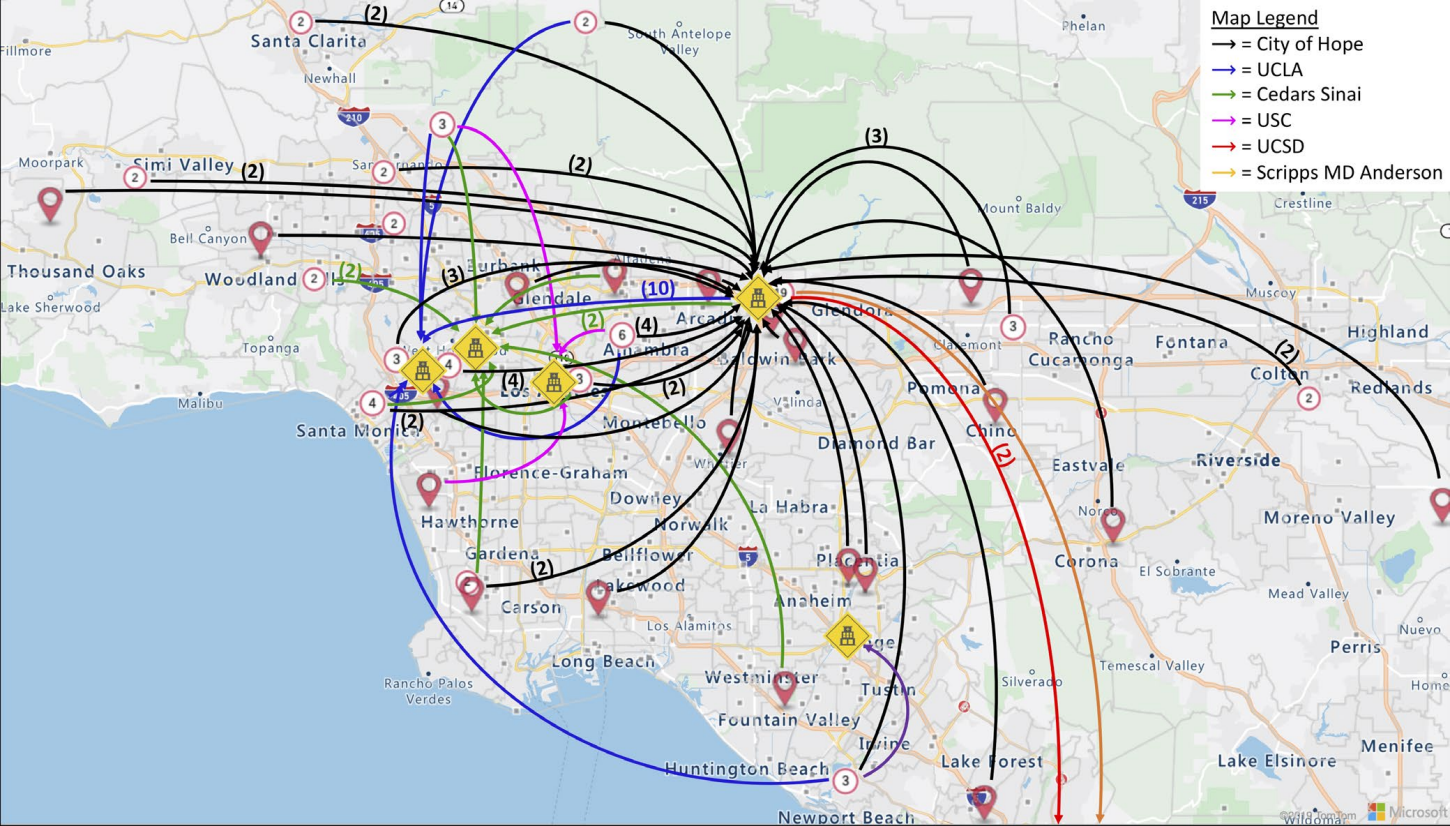
Patterns of referrals to tertiary centers in Southern California. Survey participants were asked to which Southern California tertiary cancer center they most frequently refer their patients (if based at a tertiary center, participants were instructed to select a site outside their own). Participants are mapped geographically by the zip code of their practice (red pin =single respondent, red circle =cluster of respondents; the encircled numerical value denotes the number of respondents within the cluster). Colored arrows indicate referrals from practitioners to tertiary centers; multiple instances of the same referral pathway are denoted in parentheses. UCLA, University of California, Los Angeles; USC, University of Southern California; UCSD, University of California, San Diego

All respondents were queried to which Southern California tertiary cancer center they most frequently refer patients; tertiary providers were asked to exclude any referrals to their home institutions. Across both cohorts, most respondents (46%) referred 1–5 patients to tertiary centers, and nearly all (94%) referred at least 1 patient per year. Of respondents who referred at least 1 patient annually to tertiary centers, 50 were based in the community and 30 were based in tertiary centers themselves. The average distance between the referring provider and their most commonly referred to tertiary center was 27.5 miles (range: 3.5–97.1 miles) (Figure 1). The most common reason for referral to a tertiary center was clinical trial availability (73%) followed by physician expertise (16%). Of the choices offered, a referral to a tertiary center was least frequently provided for reasons of patient requests (5%) or patient transportation needs (4%). Physicians cited financial considerations (59%) as the biggest barrier to having their patients seen at tertiary centers. Forty‐eight percent of community providers versus 15% of those at tertiary centers indicated that insurance was a factor when deciding referrals to tertiary centers (*p* = 0.003). When providers did refer patients to a specialist, 88% contacted the specialist ahead of the consult, most frequently through email (72%).

### 3.3 Clinical trials and precision medicine

Sixty‐seven providers offered clinical trials within their current practice––the proportion of physicians offering clinical trials was higher at tertiary centers versus community practices (97% and 67%, respectively; *p* = 0.001). Most physicians (82%) felt that they had a minimal‐to‐moderate understanding of clinical trials being offered by tertiary centers in their area. Furthermore, most oncologists referred patients for clinical trials if they had advanced, treatment‐refractory disease (94%)––it was uncommon to refer patients if they had early stage disease (4%) or advanced, treatment‐naïve disease (2%). Patients were most commonly referred for consideration of phase I clinical trials (44%), followed by phase II (32%) and phase III (24%). This differed among physicians based at tertiary and community centers––the former were more likely to refer for phase I trials (61% vs. 31%; *p* = 0.01). Physicians across both community and tertiary practice settings (82%) reported high comfort in referring patients to tertiary providers for clinical trial considerations.

### 3.4 Strategies for knowledge acquisition

**FIGURE 2:**
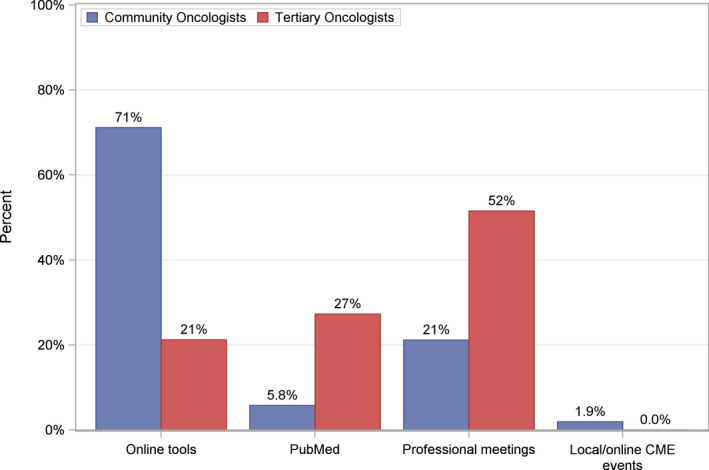
Strategies for knowledge acquisition among medical oncologists. Preferred strategies for acquiring clinical information, including up‐to‐date guidelines and best clinical practices, among tertiary and community oncologists in Southern California. Clinicians were directed to select only one strategy within the survey prompt

**FIGURE 3:**
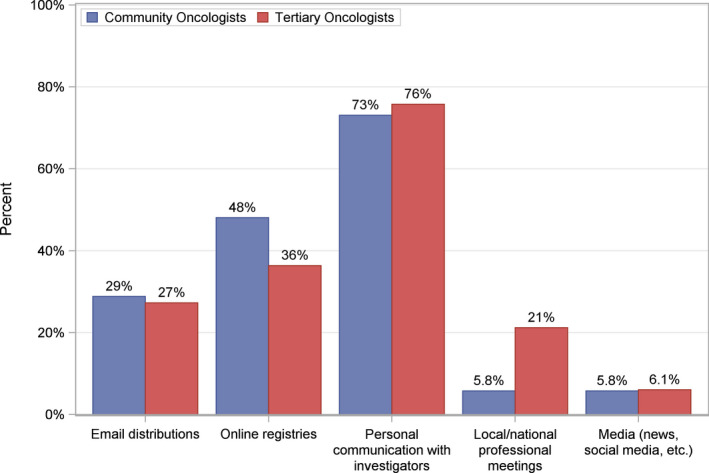
Strategies for learning about clinical trials in Southern California. Clinicians’ strategies for acquiring information on clinical trial availability in Southern California among tertiary and community oncologists. Clinicians were directed to select all strategies that applied within the survey prompt

Physicians in the community were most likely to rely on online tools such as PracticeUpdate®, UpToDate©, and OncologyTube© (71%) for acquiring knowledge regarding novel therapies and clinical research. In contrast, physicians in tertiary centers relied mostly on professional meetings such as the American Society of Clinical Oncology and European Society for Medical Oncology annual meetings (52%) (Figure 2). The preferred avenue for learning about clinical trial offerings was personal communication with investigators (74%), online registries (44%), and email distributions (28%) (Figure 3). Professional meetings (12%) and media sources (including social media) (6%) were the least preferred means of receiving information about clinical trials.

### 3.5 Perceptions of value in tertiary–community practice relations

The perceived value provided by both community and tertiary oncologists was interrogated through open‐ended questions at the end of the survey. The value of community practitioners was centered on the volume of patients seen, ease of access for patients, and source of referrals for clinical trials at tertiary centers. In contrast, the primary values of tertiary practices were focused on physician expertise, clinical trial availability, and access to novel therapeutics. Of note, a majority of tertiary center providers (52%) described the primary value of community practices to be a source of referrals for clinical trials.

## 4 DISCUSSION

These results offer a first effort to characterize the clinical and academic practices of tertiary and community oncologists using a cohort based across multiple care networks in Southern California. Our findings highlight a relative paucity of clinical trial options among community oncology practices; this alongside barriers addressed in previous publications suggest that community practitioners refer patients less often to clinical trials compared to their colleagues at tertiary centers.^5^, ^6^, ^7^, ^8^, ^9^ Our study also expands upon the current state of community–tertiary provider relations while examining novel barriers that may contribute to referral patterns for clinical trial enrollment and specialized care.

At the conclusion of the survey, providers were asked open‐ended questions about their insights and experiences with tertiary–community practice relations. With respect to strategies for better integrating practices, three common themes were prevailed: improved communication for referral cases, better advertisement of clinical trial availability, and standardized insurance contracting across networks. Respondents suggested that the development of publicly accessible clinical trial databases, routine distribution of newsletters highlighting trial availability, and educational programs focused on available clinical trials could be utilized to better increase the awareness of clinical trial offerings.

Increased follow‐up communication about referred patients was cited as a cornerstone for improving community–tertiary provider relationships. While 87% of community practitioners established contact with specialists prior to referring patients, the former raised concerns about the delay for consults and subsequent lack of return communication regarding clinical decisions. Deficiencies in bidirectional communication likely have negative consequences for the patient––it has been shown that such disruptions of communication during transitions of care increase the risk of substantial clinical morbidity and worsened patient outcomes.^11^, ^12^

Communication surrounding clinical trials also was reported as an area for improvement in tertiary–community oncology relations. The majority of physicians surveyed (82%) indicated only a minimal‐to‐moderate understanding of clinical trials being offered at tertiary cancer centers in Southern California. Additionally, we report that most oncologists (74%) relied on direct communication with investigators to learn of clinical trial offerings, regardless of practice setting. The current model of clinical trial communication and transparency may, therefore, be a leading impediment in referral volume and trial enrollment. As reflected in our survey, nearly half of community oncologists routinely refer only 1–5 patients per year to tertiary centers, primarily for clinical trial considerations. Yet, a majority of tertiary providers (52%) asserted the primary value of community practices was to be a source of clinical trial referrals. Previous work has demonstrated that improved clinical trial education for staff contributed to increased trial enrollment within a single tertiary cancer center, which, implemented across care networks and settings, may prove useful in increasing trial‐specific referrals.^13^ If more effective tools for clinical trial information dissemination and communication were established, such as staff education efforts and the previously mentioned publicly accessible databases, we hypothesize this would further increase both referral volume and clinical trial enrollment across Southern California.

Differences in insurance contracting across networks were also referenced as a target for improved practice integration. Indeed, financial considerations were cited as the biggest barrier to patient referrals by survey respondents (59%). Work from Warsame and colleagues has detailed the prevalence of financial discussions in oncology care, noting that approximately one in four patient visits include discussion of financial issues.^14^ These discussions often guided medical decision making and clinical trial enrollment. As healthcare costs continue to rise, particularly in oncology, exploration into the deterministic nature of patient finances and insurance status in clinical decision making and referrals is warranted.^15^

Recent work has further defined gaps in patient outcomes between the tertiary and community oncology settings. Boffa *et al* examined perioperative mortality and long‐term survival—key surgical endpoints for cancer patients—at top‐ranked tertiary centers and affiliated community sites.^16^ This retrospective analysis of nearly 120,000 patient cases (33% of whom were treated at affiliated community centers) concluded that perioperative mortality was higher at affiliate centers than at the tertiary centers and that long‐term survival was significantly lower at affiliate practices. Additional work from Syed *et al* has demonstrated that length of stay for certain urologic cancer surgeries was shorter in the tertiary setting as compared to community practice.^17^ These studies are both in the domain of surgical oncology, while our study is medical oncology focused. While we do not directly interrogate patient outcomes between tertiary and community sites, differences in utility of genomic profiling platforms and availability of clinical trials between the settings were noted in our survey responses, which may influence clinical outcomes.^18^, ^19^ As future work, we plan to track outcomes longitudinally in patients receiving care at our tertiary center versus affiliated community practices.

The shifting paradigms of healthcare delivery have recently led to network consolidation, in which large tertiary centers are increasingly establishing partnerships with community practice clinics. A majority (60%) of medical oncologists who completed this survey were affiliates of a hybrid academic‐community practice model through the City of Hope Comprehensive Cancer Center. The City of Hope network consists of a tertiary center, designated as an NCI‐CCC, and 30 community practice sites within a 100 mile radius in Southern California. Recent reports from physicians in the City of Hope network have highlighted the efficiencies and limitations of this care model.^20^, ^21^, ^22^, ^23^, ^24^, ^25^, ^26^ These pieces noted increased trial accrual, implementation of cancer screening programs, and integration of precision medicine efforts, among other collaborative measures, at affiliated community sites as favorable consequences of the tertiary–community partnership. The City of Hope model and similar partnerships nationwide may potentially ameliorate differences in oncology practice and outcomes between tertiary and community sites discussed in this study and other referenced publications.^27^

This survey protocol has multiple limitations that may affect the generalizability of these results. First, participants invited to this study were chosen from a highly selected group of individuals, primarily through professional contacts. This was in part done to ensure high participation rates. Indeed, 87% of individuals who received an invitation completed the study in full, which exceeds similar survey protocols. Additionally, this work was carried out only among providers in Southern California––a region with a dense population of tertiary cancer centers. These relationships reported herein may not be representative of other geographic regions with less access to tertiary cancer care. Although there were over a dozen care networks represented in this protocol, the majority of respondents were members of the City of Hope network. This affiliation limits the heterogeneity of care networks and settings. To enhance the generalizability of our results, an important measure would be to utilize national databases that characterize more fully practice patterns in different geographical areas. This work is being actively pursued.

This survey provides key insights into the current state of tertiary–community oncology practice relations in Southern California. Providers highlighted communication surrounding patient referrals and clinical trials, as well as financial considerations, as the biggest constraints to community–tertiary relationships. While differences in clinical practice are reported across the practice settings, a focus on strategies to advance and synergize partnerships between community and tertiary centers may provide opportunities to improve practice relations and, in turn, patient outcomes.

## ETHICAL APPROVAL STATEMENT

This study's protocol was reviewed and approved by the City of Hope Comprehensive Cancer Center Institutional Review Board. All subjects provided informed consent prior to participation.

## CONFLICT OF INTEREST

Nicholas Salgia, Alexander Chehrazi‐Raffle, JoAnn Hsu, Zeynep Zengin, Sabrina Salgia, Neal Chawla, Luis Meza, Jasnoor Malhotra, Ramya Muddasani, Nora Ruel, Jun Gong, Sidharth Anand, Victor Chiu, James Yeh, and Mary Cianfrocca declare that they have no conflict of interest that might be relevant to the contents of this manuscript. Nazli Dizman, MD: Consulting Role––Vivreon Bioscience. Sumanta K. Pal, MD: Honoraria––Novartis, Medivation, Astellas Pharma; Consulting or Advisory Role––Pfizer, Novartis, Aveo, Myriad, Pharmaceuticals, Genentech, Exelixis, Bristol‐Myers Squibb, Astellas Pharma; Research Funding––Medivation.

## AUTHOR CONTRIBUTIONS

Conceptualization: NJS, ACR, SKP, JH, and MC; Data Curation: NJS, ACR, JG, SA, VC, and JY; Formal Analysis: NJS, ACR, and NR; Funding Acquisition: N/A; Methodology: NJS, ACR, and SKP; Project Administration: NJS, ACR, SKP, and JH; Resources: SKP and JH; Software: N/A; Supervision: SKP; Validation: NJS, ACR, and NR; Visualization: NJS, ACR, and NR; Writing––Original Draft: NJS and ACR; Writing––Review and Editing: NJS, ACR, JH, ZZ, SS, NSC, LM, JM, RM, ND, NR, JG, SA, VC, JY, MC, and SKP.

## Supporting information

Appendix S1

## Data Availability

The data that support the findings of this study are available from the corresponding author, SKP, upon reasonable request.
