# Supplementary material for: Characterizing the relationships between tertiary and community cancer providers: Results from a survey of medical oncologists in Southern California
Source: Cancer Med. 2021 Jul 31;10(16):5671–80. doi: 10.1002/cam4.4119 (PMC8366095; doi:10.1002/cam4.4119)
Supplement: Supplementary file 1 — Appendix S1 [file CAM4-10-5671-s001.pdf]

Clinical Trial Referral Survey

Thank you for your participation!

1) What is your email address?

____

2) What is the zip code of your practice?

____

3) What is your age?

- ◯ 21-30
- ◯ 31-40
- ◯ 41-50
- ◯ 51-60
- ◯ 61-70
- ◯ 71-80
- ◯ 80+

4) What is your gender

- ◯ Male
- ◯ Female
- ◯ Other

5) In what practice setting do you primarily work in currently?

- ◯ Tertiary care center
- ◯ Inpatient oncology at a community hospital
- ◯ Outpatient community practice affiliated with a tertiary center
- ◯ Outpatient community practice/private practice not affiliated with a tertiary center
- ◯ Veterans Affairs or other government/military practice

6) How many years have you been a medical oncology attending in clinical practice?

- ◯ 0-5
- ◯ 6-10
- ◯ 11-15
- ◯ 16-20
- ◯ 21-25
- ◯ 26-30
- ◯ 30+

7) Which tertiary cancer center (if any) is your practice affiliated with?

- ◯ Cedars-Sinai
- ◯ City of Hope
- ◯ Scripps MDAnderson San Diego
- ◯ UC Irvine
- ◯ UCLA
- ◯ UC San Diego
- ◯ USC
- ◯ I am not affiliated with a tertiary cancer center

8) How long have you been affiliated with your current practice/institution?

- ◯ 0-5 years
- ◯ 6-10 years
- ◯ 11-15 years
- ◯ 16-20 years
- ◯ 21+ years

9) Do you have a particular focus within oncology?

- ◯ Breast medical oncology
- ◯ Gastrointestinal medical oncology
- ◯ General medical oncology
- ◯ Genitourinary medical oncology
- ◯ Gynecologic medical oncology
- ◯ Head and neck medical oncology
- ◯ Radiation oncology
- ◯ Surgical oncology
- ◯ Thoracic medical oncology
- ◯ Other (sarcomas, melanoma, neuro, etc.)

---

10) Which area do you consider having the weakest understanding of within oncology?

- ◯ Breast oncology
- ◯ CNS oncology
- ◯ Dermatologic oncology
- ◯ Gastrointestinal oncology
- ◯ Genitourinary oncology
- ◯ Gynecologic oncology
- ◯ Head and neck oncology
- ◯ Sarcoma
- ◯ Thoracic oncology

---

11) Which resource do you rely on most to learn of the most up-to-date clinical research and advancements in oncology, including therapeutic options and practice guidelines?

- ◯ Professional meetings (i.e. ASCO, ESMO, etc.)
- ◯ PubMed or other literature databases
- ◯ Local or online CME events (not including large conferences or professional meetings)
- ◯ Online tools (i.e. PracticeUpdate, UpToDate, OncologyTube, etc.)

---

12) How many patients do you see per week in your clinic?

- ◯ 0-10
- ◯ 11-20
- ◯ 21-40
- ◯ 41-60
- ◯ 61-80
- ◯ 81+

---

13) What insurances do you accept (check all that apply)?

- ☐ PPO
- ☐ HMO
- ☐ Medi-Cal
- ☐ Medicare
- ☐ VA

---

14) Do you factor in insurance status when deciding to refer to a tertiary center?

- ◯ Yes
- ◯ No

---

15) Are you offering clinical trials at your practice?

- ◯ Yes
- ◯ No

---

16) How well do you feel you know what clinical trials are being offered at Southern California tertiary centers?

- ◯ Very well
- ◯ Moderately well
- ◯ Minimally well
- ◯ Not at all

17) How do you learn about clinical trials offered at Southern California tertiary centers?

- ☐ E-mail distributions
- ☐ Online registries (e.g., CTOL)
- ☐ Personal communication with tertiary campus physicians
- ☐ Local or national professional meetings
- ☐ Social media (Twitter, Facebook, LinkedIn, etc.)
- ☐ News/other forms of media (e.g. press releases, blog posts, news articles, etc.)

18) Do you offer genomic profiling to your patients? If yes, which platform do you use most frequently?

- ◯ No I do not offer genomic testing
- ◯ Ashion GEM Extra
- ◯ Caris
- ◯ FoundationOne
- ◯ Guardant360
- ◯ Tempus
- ◯ Other

19) How frequently has genomic information guided a patient of yours to a clinical trial?

- ◯ < 1%
- ◯ 1-10%
- ◯ 11-25%
- ◯ 26-50%
- ◯ 51%+

20) How many patients do you refer per year to tertiary centers? (If you work at a tertiary center, note how many patients you refer to other tertiary centers.)

- ◯ 0
- ◯ 1-5
- ◯ 6-10
- ◯ 11-15
- ◯ 16-20
- ◯ 21+

21) Which tertiary center do you refer to most frequently? (If you work at one of the listed tertiary centers, note which site outside of your own that you refer to most often.)

- ◯ Cedars Sinai
- ◯ City of Hope
- ◯ Scripps MDAnderson San Diego
- ◯ UCLA
- ◯ USC
- ◯ UC Irvine
- ◯ UC San Diego

22) What is your primary reason for referring to (other) tertiary centers?

- ◯ Clinical trial available at specific institution
- ◯ Patient transportation needs
- ◯ Patient requests
- ◯ Physician expertise

23) What is the biggest barrier to getting your patients seen at (other) tertiary centers?

- ◯ Transportation to tertiary campus
- ◯ Lengthy wait times for providers
- ◯ Financial considerations (e.g., insurance contracts)

24) Do you often get in touch with providers at (other) tertiary centers ahead of a second opinion appointment for your patient? If so, how do you primarily communicate?

- ◯ No I do not contact other providers ahead of referrals
- ◯ Phone call
- ◯ Pager
- ◯ E-mail
- ◯ Text message
- ◯ EMR messaging

25) How would you characterize the majority of patients you refer to (other) tertiary centers?

- ◯ Early stage disease, possible consideration for adjuvant/neoadjuvant therapy
- ◯ Advanced disease, treatment-naïve
- ◯ Advanced disease, treatment-refractory

26) If you refer to (other) tertiary centers, for what study phase do you most often refer?

- ◯ Phase I
- ◯ Phase II
- ◯ Phase III

27) On a scale of 1-10, how comfortable are you referring patients to (other) tertiary centers? (1=not at all comfortable, 10=extremely comfortable)?

- ◯ 1
- ◯ 2
- ◯ 3
- ◯ 4
- ◯ 5
- ◯ 6
- ◯ 7
- ◯ 8
- ◯ 9
- ◯ 10

28) What are some ways tertiary centers can better integrate the community with main campus?

____

29) What value do you think the community practice provides to tertiary centers?

____

30) What value do you think tertiary centers provide to community practices?

____
